# Supplementary material for: Modeling the 2022 mpox outbreak with a mechanistic network model
Source: BMC Infect Dis. 2025 Nov 5;25:1507. doi: 10.1186/s12879-025-11731-7 (PMC12587546; doi:10.1186/s12879-025-11731-7)
Supplement: Supplementary file 1 — Supplementary Material 1. [file 12879_2025_11731_MOESM1_ESM.pdf]

1 Supplementary Information

1.1 Vaccination Information

The number of vaccines administered in the modeling reflects the number of first and second doses of JYNNEOS vaccines received in the US from May 22, 2022 to July 1, 2023. The raw data was obtained on July 11, 2023 from the CDC's website[1]. To account for the fact that not everyone who received a vaccine was male, the number of first doses given was multiplied by 0.91, as 91% of those who received a first dose were male, and the number of second doses was multiplied by 0.94 for the same reason, per the CDC's information on vaccine administration. To account for population, we divided the number of doses by the total population at risk, 1,998,039[2, 3]. Therefore, we were able to approximate the number of vaccines available in a given week in a population of 10,000 individuals per the national averages. Daily numbers were obtained by dividing the weekly numbers by 7. Figure S1 shows the number of vaccines available in our simulation per day by dose type.

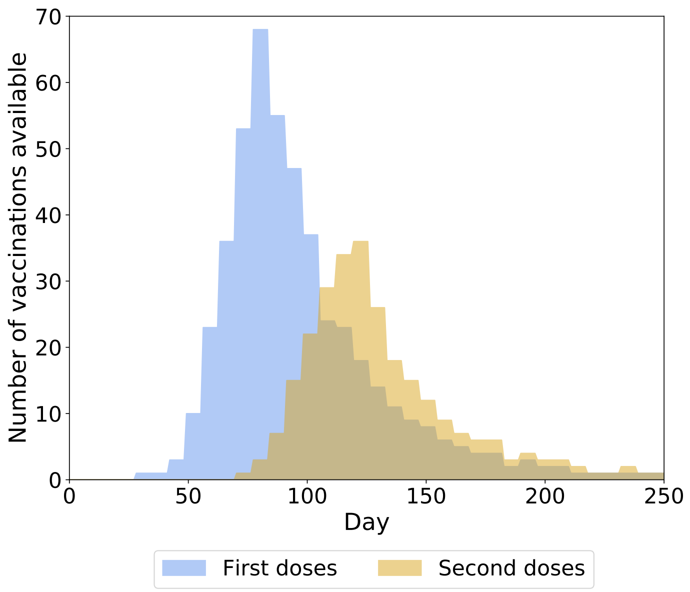

Fig. S1 Vaccine availability by day and dose type.

1.2 Sensitivity Analyses

1.2.1 Vaccine Efficacy

Our primary results assume that vaccine efficacy is 33% for the first dose and 66% for full vaccination, per the results from Deputy et al. [4]. However, a meta-analysis by Pischel et al. estimated vaccine efficacy as 76% for a single dose and 82% for full vaccination. As a sensitivity analysis, we simulated the effect of higher vaccine efficacy over a range of outbreak mitigation strategy timings in Figure S2. As expected, the simulations with a more efficacious vaccine resulted in fewer infections over 250 days.

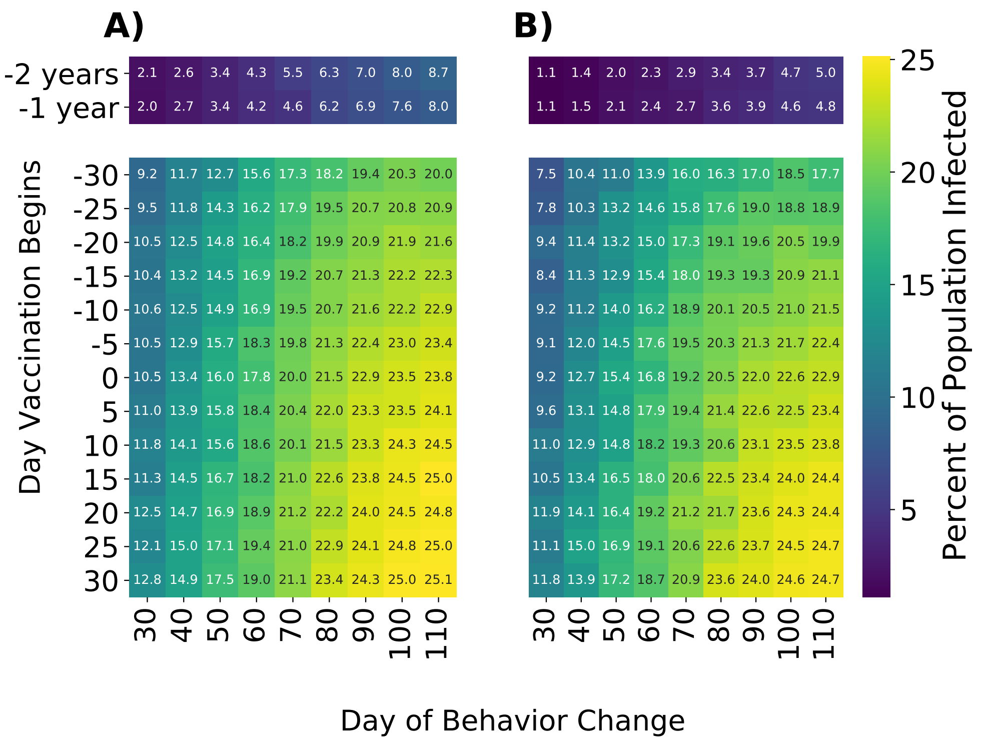

Fig. S2 Percent of the population infected with mpox after 250 days under different outbreak mitigation strategy timings and with different vaccine efficacies. Strategies only affect men in strata 5 and 6 of sexual activity. All simulations assume individuals reduce their probability of having a one-time partner by 50%. Panel A shows results from simulations with vaccine efficacy from the Deputy et al. study [4]; Panel B shows results from simulations with vaccine efficacy from the Pischel et al. study[5]. Cell values indicate the percent of the network infected after 250 days. Rows indicate the day that vaccines become available; negative numbers indicate vaccination becoming available prior to the start of the outbreak.

1.2.2 Isolation Compliance

As a sensitivity analysis, we also present the results comparing the percentage of the population that becomes infected after 250 days if we assume a less optimistic isolation

scheme in which, once diagnosed, individuals only fully isolate from one-time partnerships but partially isolate from main and casual partnerships. Comparing Figure S3 to the main results in Figure 3, partial compliance leads to a greater number of overall infections but does not change the conclusion that behavioral change and vaccination reduce the final size of the outbreak. Without outbreak mitigation strategies and with only partial compliance the 26.42% of the population is infected after 250 days, ($P_{25\%}$ and $P_{75\%}$: 25.67%, 27.15%). With universal vaccination and behavior adaptions, this decreases to 21.04% ($P_{25\%}$ and $P_{75\%}$: 19.53%, 23.01%).

Figure S4 shows the results at different outbreak mitigation strategy timings and intensities with partial isolation compliance (Panel A) and full compliance (Panel B). Panel B contains the same information as Figure 6, but the colors are recalculated to include the entire range in Panel A. We can see that greater isolation compliance (Panel B) can be as protective at the population level as more intense and earlier vaccination and behavior adaptation (Figure S4). For example, full isolation compliance with vaccination beginning on day 30 and 50% behavior change beginning on day 70 (11.7% of the population infected) is similar to partial isolation compliance, but with more intense behavior change, and both outbreak mitigation strategies beginning 20 days earlier.

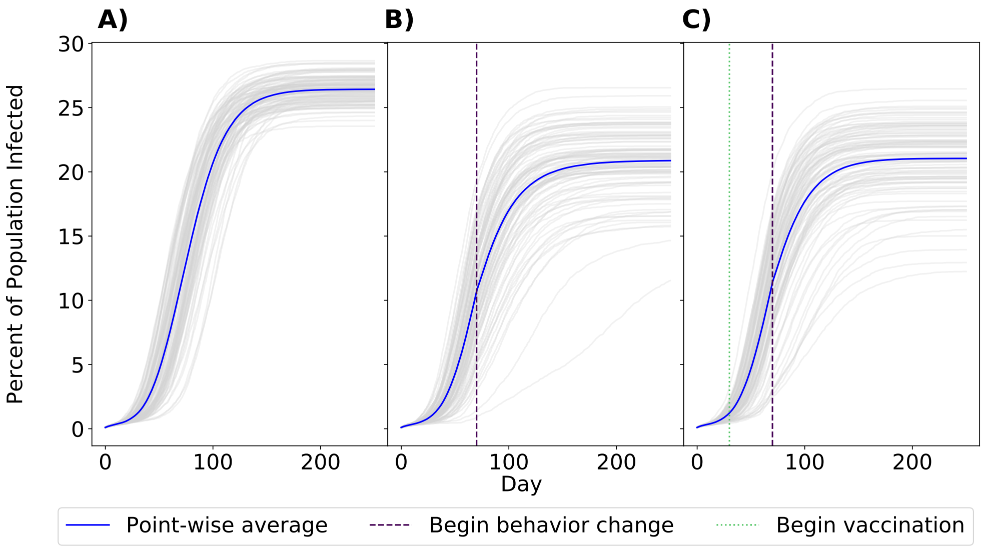

Fig. S3 Comparison of universal outbreak mitigation strategies with partial isolation compliance. Panels indicate the percent of the network infected with mpox after 250 days with no outbreak mitigation strategy (Panel A), universal behavior change (Panel B), or universal behavior change with vaccination (Panel C). Grey lines denote individual simulations. The point-wise average is shown in blue. Vertical lines indicate the day of outbreak mitigation strategy initiation.

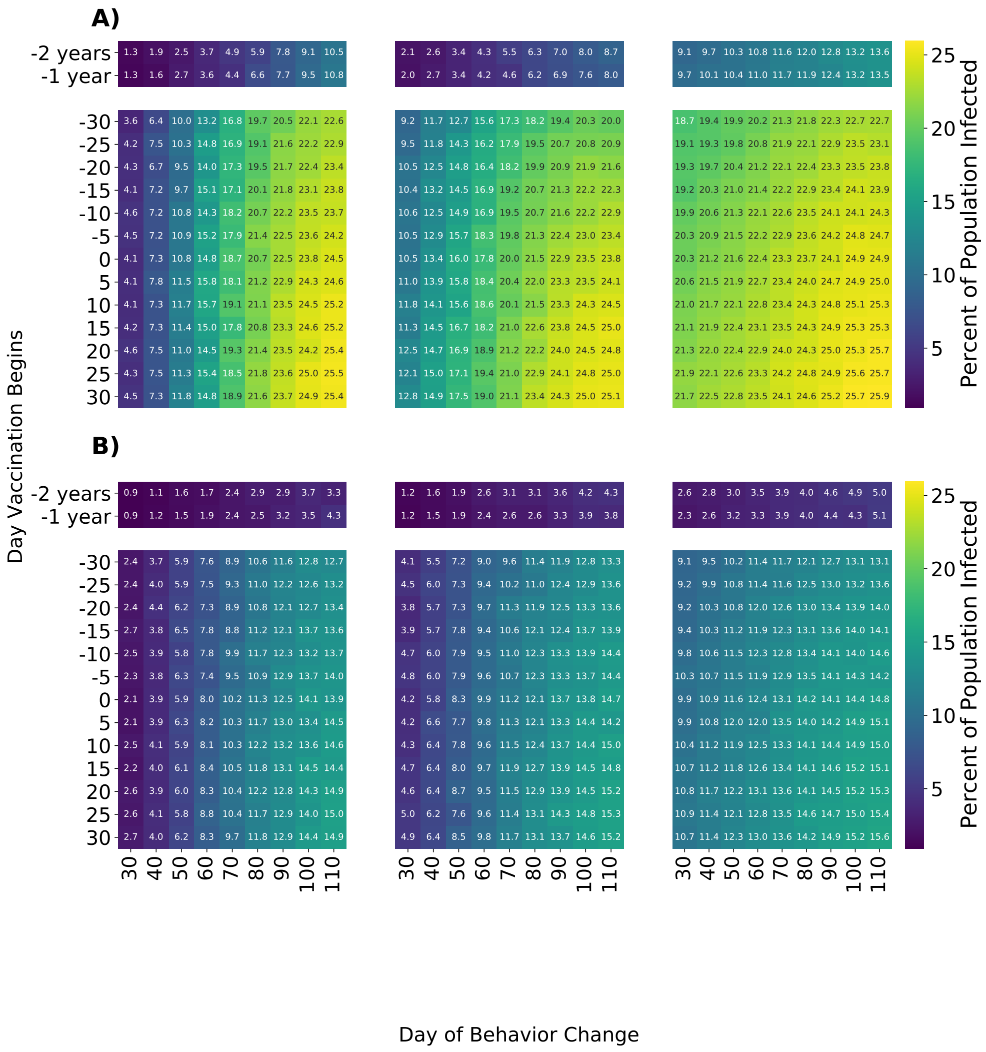

Fig. S4 Percent of the population infected with mpox after 250 days under different outbreak mitigation strategy timings and intensities and with different levels of isolation compliance. Vaccination and behavior adaptation only affect men in strata 5 and 6 of sexual activity. Panel A shows results from simulations with partial isolation compliance; Panel B shows results from simulations with full isolation compliance. Cell values indicate the percent of the network infected after 250 days. Rows indicate the day that vaccines become available; negative numbers indicate vaccination becoming available prior to the start of the outbreak. The left, middle, and right columns show simulations where individuals reduce their probability of having a one-time partner by 75%, 50%, and 25%, respectively.

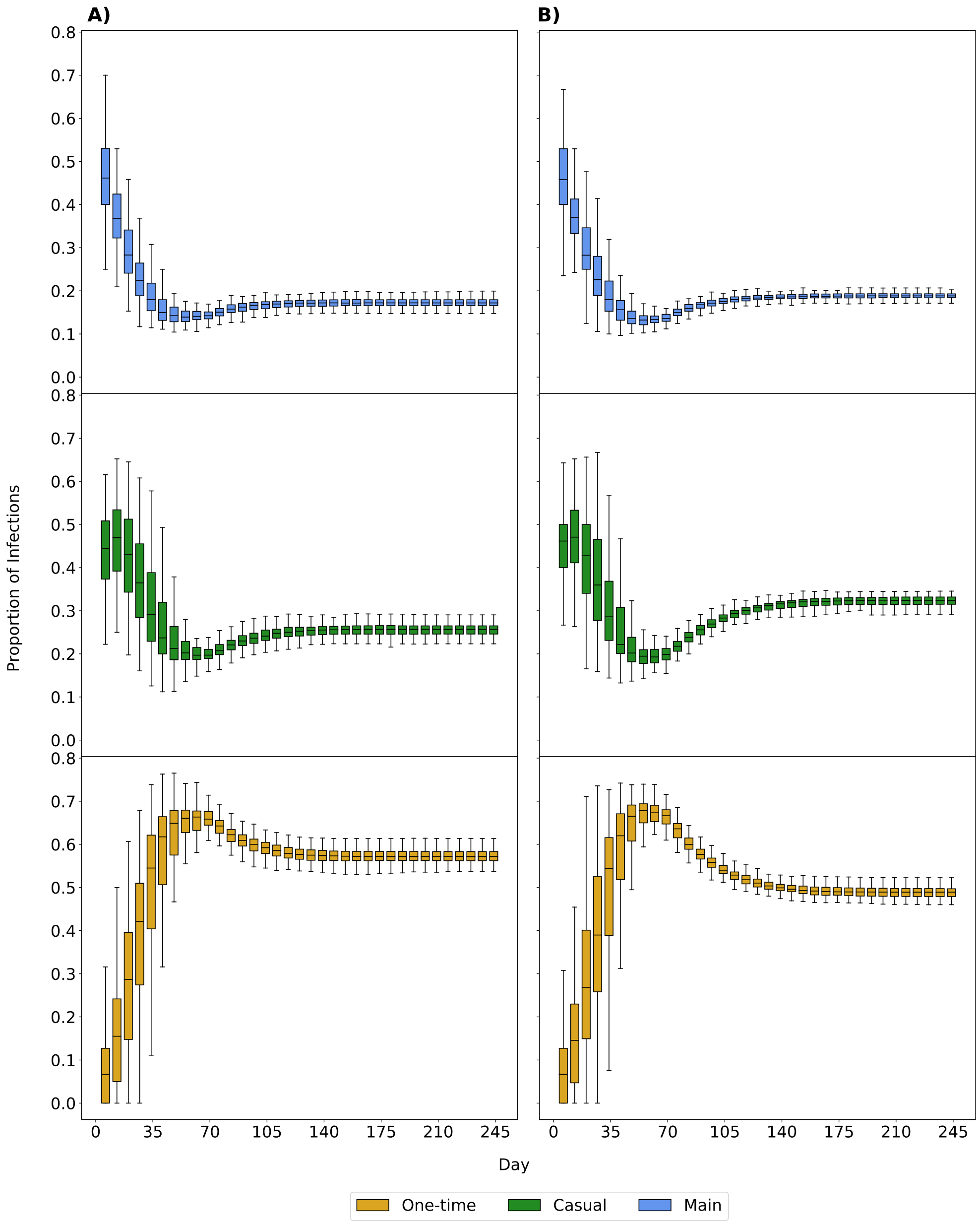

Fig. S5 Proportion of infections attributable to each relationship type. Panels indicate the proportion of cumulative infections by time t attributable to each relationship type when vaccination and behavior adaptation occur in the 25% of men most likely to have a one-time partner. Panel A shows the results with full isolation compliance after diagnosis; Panel B shows partial compliance. Box plots show results of 100 independent simulations presented for every 7 days of data.

1.2.3 Infection Parameters

Due to the uncertainty around the clinical features of mpox, particularly the length of time individuals are in the exposed and infectious states; we conducted additional sensitivity analyses to ensure our findings are robust to the range of current estimates of the infection parameters. We selected incubation times from a review of published infection-to-onset times, which corrected for right-truncation bias[6], and for the length of time individuals are infectious, we use the standard 2-4 week range. We created two scenarios: one which used the estimates that were most likely to lead to few infections that had a long incubation period and a short infection period (5.6 days and 2 weeks, respectively), the optimistic scenario, and one which used estimates most likely to lead to many infections that had a short incubation period and a long infection period (9.9 days and 4 weeks, respectively), the pessimistic scenario. Figure S6 shows the results of these scenarios, with the same outbreak mitigation strategies as the main results (reduction of one-time partners at 70 days and vaccination at 30 days). As expected, we see that the optimistic scenario leads to fewer total infections than our main results (mean percent of population infected without vaccination or behavior adaptation: 11.55%, 25th and 75th percentiles of infections: 11.05%, 12.01%; mean with vaccination and behavior adaptation: 7.98%, 25th and 75th percentiles of infections: 5.03%, 10.67%), while the worst-case scenario yields more infections (mean percent of population infected without vaccination or behavior adaptation: 16.60%, 25th and 75th percentiles of infections: 15.90%, 17.20%; mean with vaccination and behavior adaptation: 18.95%, 25th and 75th percentiles of infections: 14.43%, 24.50%). However, in both scenarios, we do see that the outbreak mitigation strategies reduce or do not impact the number of overall infections.

Figures S7, S8, S9, and S10 are joint sensitivity analyses showing the results of varying the infection parameters (best and worst case scenarios presented above), the probability of transmission during sexual contact, and isolation scenarios over a range

of outbreak mitigation strategy timings. These joint sensitivity analyses demonstrate that the only scenario in which the mitigation strategies do not impact cumulative infections is in simulations under the most optimistic combination of infection parameters and a low probability of transmission during sexual contact due to lack of overall transmission of mpox in the population (Figure S9 panel A). The consistent conclusions from our sensitivity analyses indicate that our results are robust even if the infection parameters vary.

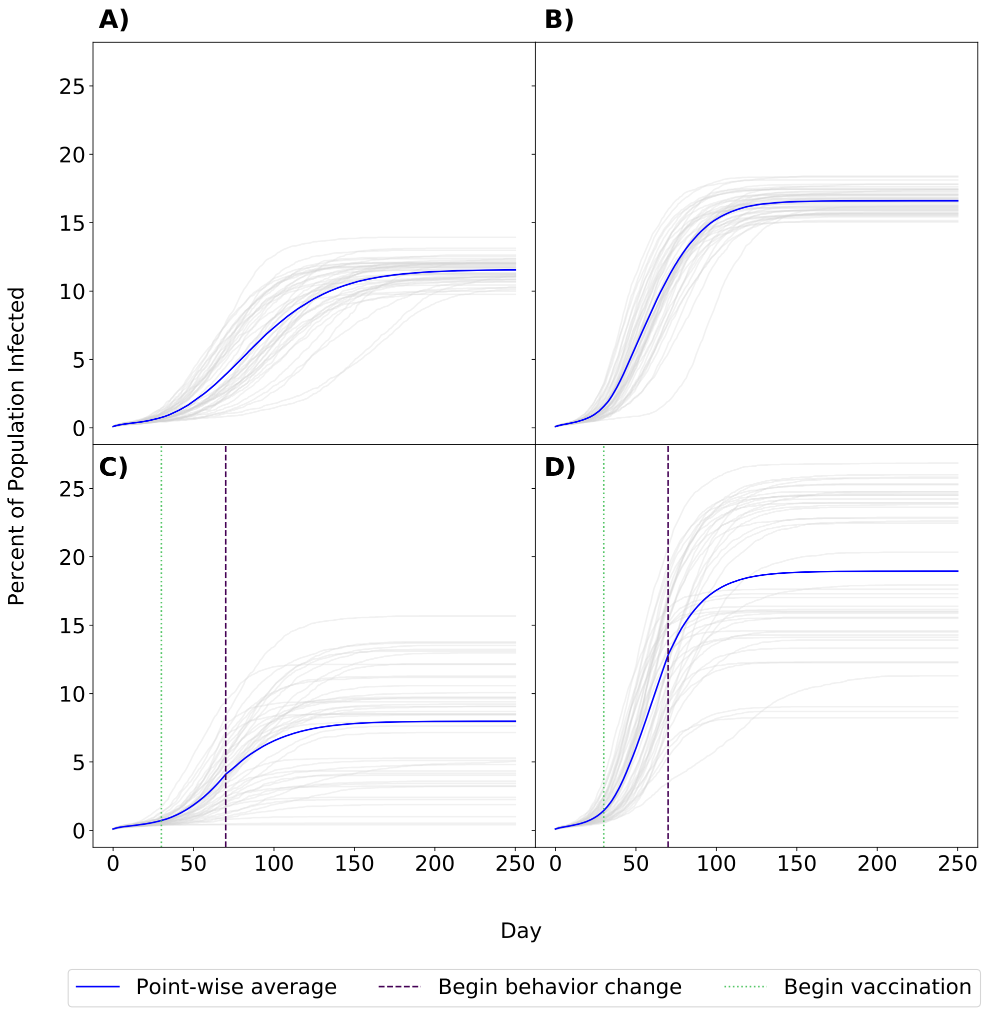

Fig. S6 Comparison of infection parameters. Panels indicate the percent of network infected with mpox after 250 days with no vaccination or behavior adaptation and optimistic infection parameters (Panel A), no vaccination or behavior adaptation and pessimistic infection parameters (Panel B), universal behavior change with vaccination and optimistic infection parameters (Panel C), or universal behavior change with vaccination and pessimistic infection parameters (Panel D). Grey lines denote 50 individual simulations. The point-wise average is shown in blue. Vertical lines indicate the day of vaccination/behavior adaptation initiation.

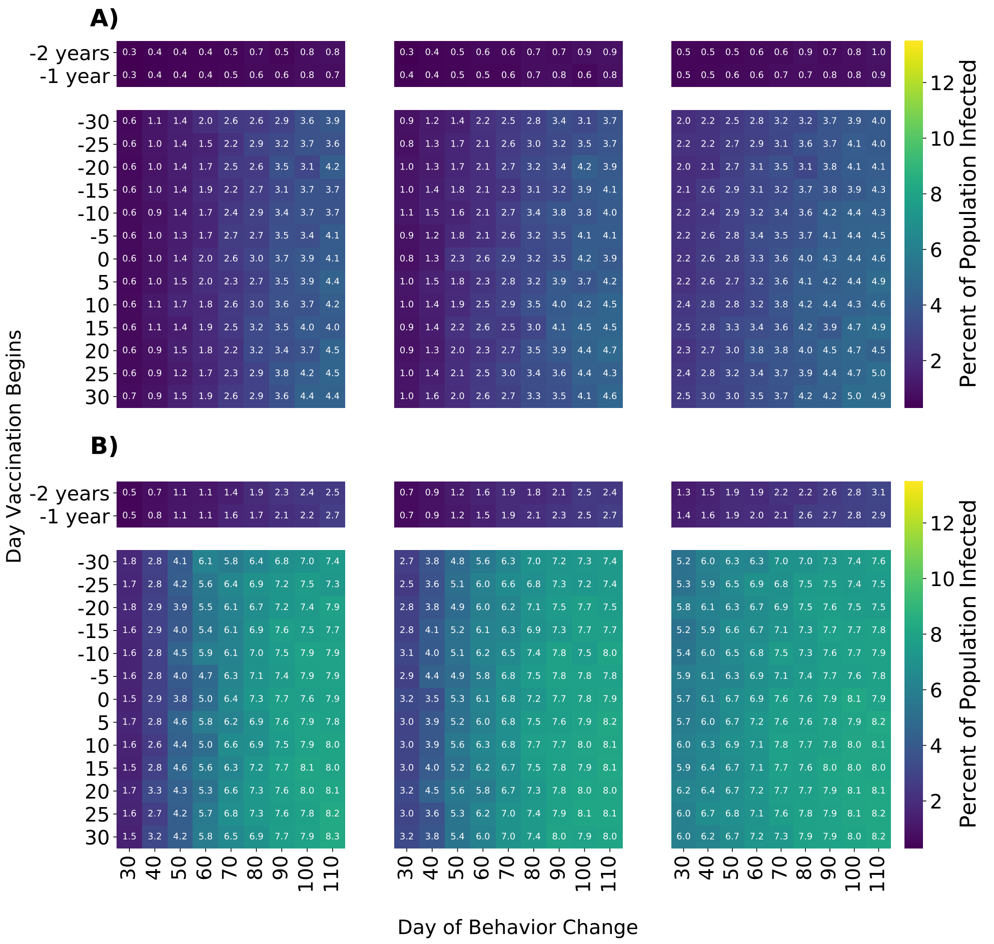

Fig. S7 Percent of the population infected with mpox after 250 days under different outbreak mitigation strategy timings and intensities and with optimistic and pessimistic infection parameters with full isolation compliance and high transmission probability. Strategies only affect GBMSM in strata 5 and 6 of sexual activity. Probability of transmission for sexual contact in a serodiscordant pair is 0.9. Panel A shows results from simulations with optimistic infection parameters; Panel B shows results from simulations with pessimistic infection parameters. Cell values indicate the percent of the network infected after 250 days. Rows indicate the day that vaccines become available; negative numbers indicate vaccination becoming available prior to the start of the outbreak. The left, middle, and right columns show simulations where individuals reduce their probability of having a one-time partner by 75%, 50%, and 25%, respectively.

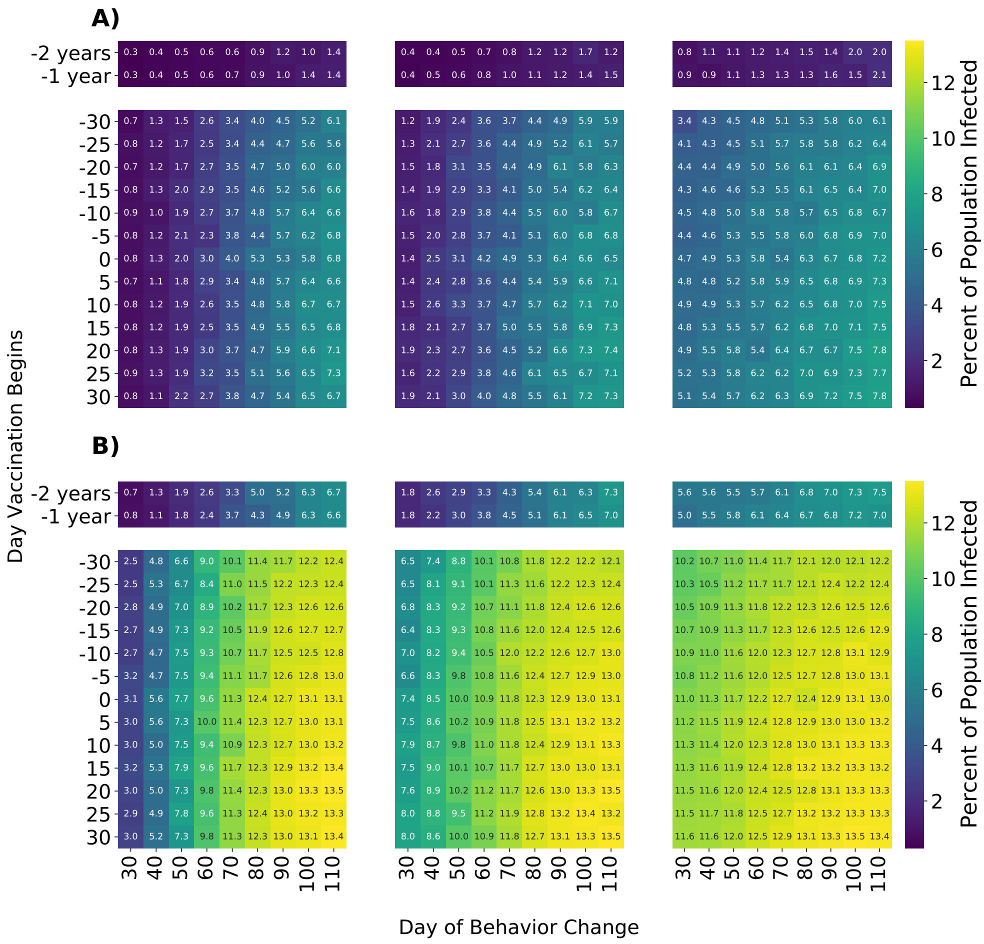

Fig. S8 Percent of the population infected with mpox after 250 days under different outbreak mitigation strategy timings and intensities and with optimistic and pessimistic infection parameters with partial isolation compliance and high transmission probability. Mitigation strategies only affect GBMSM in strata 5 and 6 of sexual activity. Probability of transmission for sexual contact in a serodiscordant pair is 0.9. Panel A shows results from simulations with optimistic infection parameters; Panel B shows results from simulations with pessimistic infection parameters. Cell values indicate the percent of the network infected after 250 days. Rows indicate the day that vaccines become available; negative numbers indicate vaccination becoming available prior to the start of the outbreak. The left, middle, and right columns show simulations where individuals reduce their probability of having a one-time partner by 75%, 50%, and 25%, respectively.

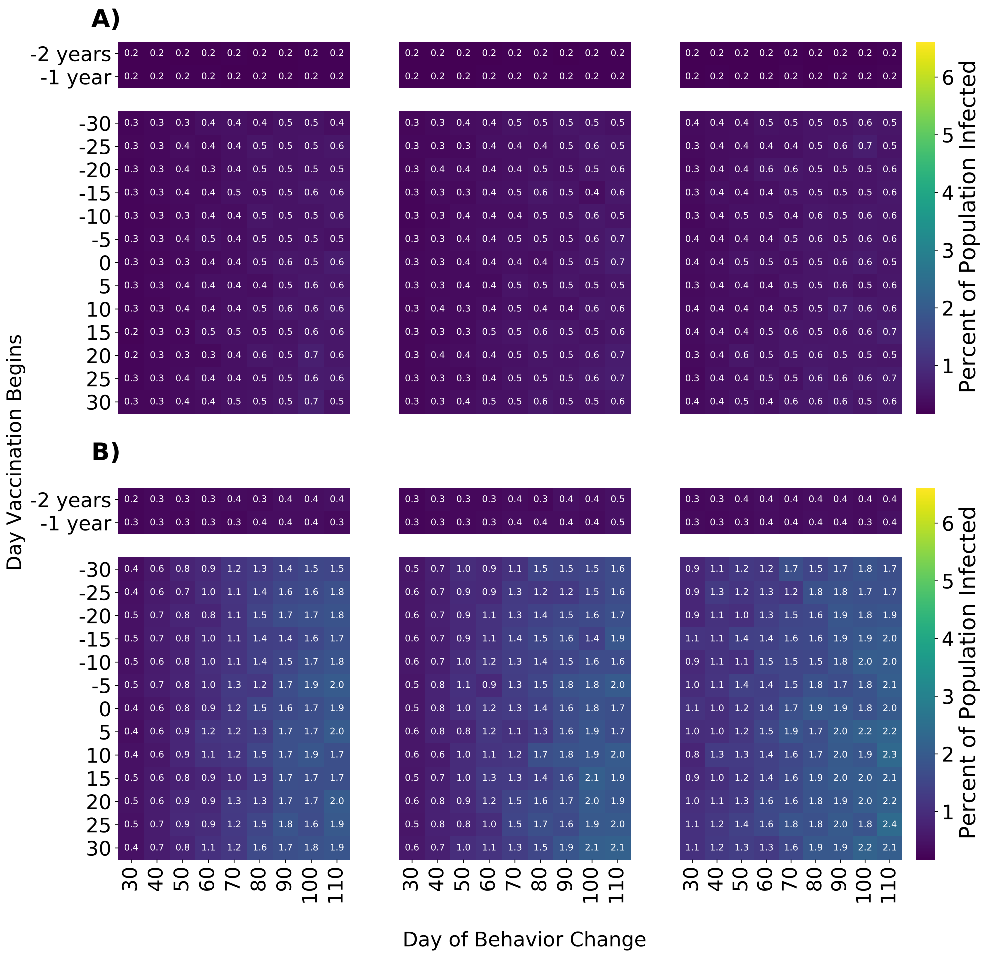

Fig. S9 Percent of the population infected with mpox after 250 days under different outbreak mitigation strategy timings and intensities and with optimistic and pessimistic infection parameters with full isolation compliance and low transmission probability. Mitigation strategies only affect GBMSM in strata 5 and 6 of sexual activity. Probability of transmission for sexual contact in a serodiscordant pair is 0.5. Panel A shows results from simulations with optimistic infection parameters; Panel B shows results from simulations with pessimistic infection parameters. Cell values indicate the percent of the network infected after 250 days. Rows indicate the day that vaccines become available; negative numbers indicate vaccination becoming available prior to the start of the outbreak. The left, middle, and right columns show simulations where individuals reduce their probability of having a one-time partner by 75%, 50%, and 25%, respectively.

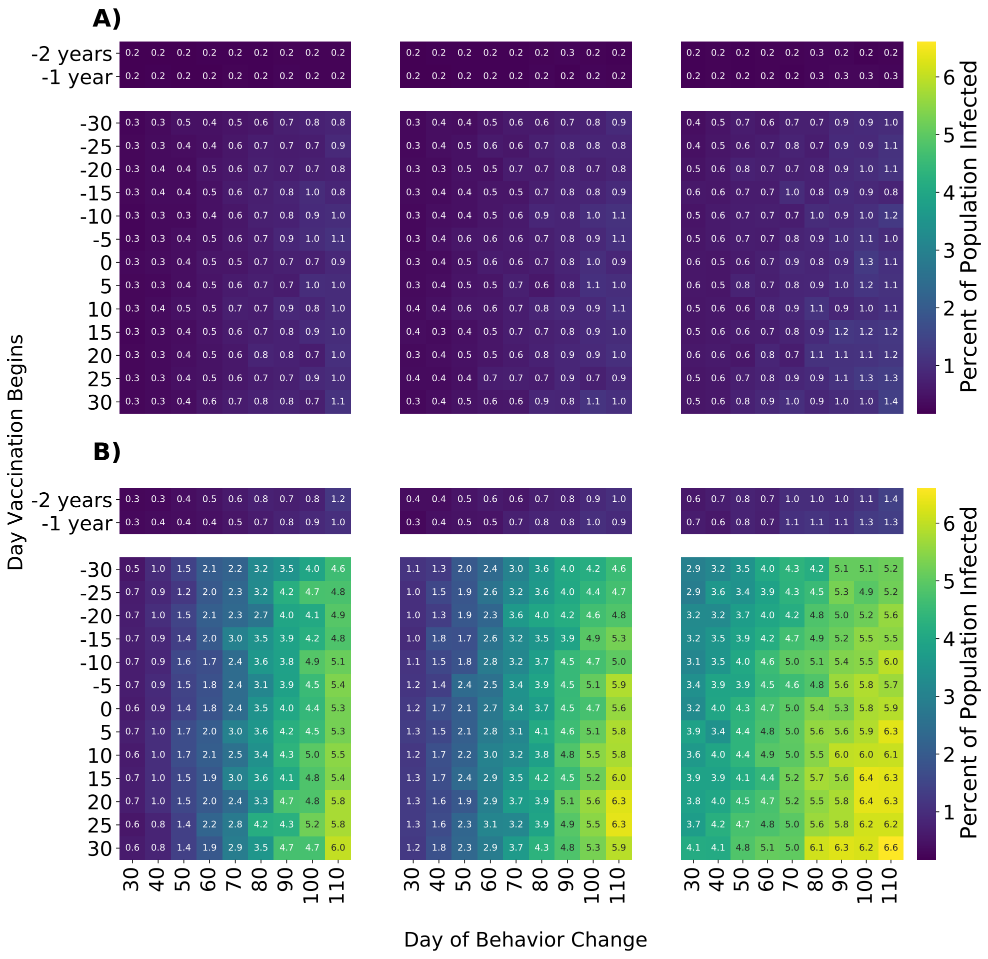

Fig. S10 Percent of the population infected with mpox after 250 days under different outbreak mitigation strategy timings and intensities and with optimistic and pessimistic infection parameters with partial isolation compliance and high transmission probability. Mitigation strategies only affect GBMSM in strata 5 and 6 of sexual activity. Probability of transmission for sexual contact in a serodiscordant pair is 0.5. Panel A shows results from simulations with optimistic infection parameters; Panel B shows results from simulations with pessimistic infection parameters. Cell values indicate the percent of the network infected after 250 days. Rows indicate the day that vaccines become available; negative numbers indicate vaccination becoming available prior to the start of the outbreak. The left, middle, and right columns show simulations where individuals reduce their probability of having a one-time partner by 75%, 50%, and 25%, respectively.

1.2.4 Population Size

To better understand the sensitivity of our results to the total population size in the simulation, we repeated the simulations for varying population sizes. Figure S11 demonstrates the results of 50 simulations for networks with N = 5,000, N = 10,000, N = 20,000, N = 40,000, and N = 80,000 nodes. The mean percent of the network infected after 250 days is consistent for different network sizes, ranging from 10.57% for the network of 5,000 nodes to 12.8% for the network of 80,000 nodes. However, the network size has a large effect on the between-simulation variability: The 25th and 75th percentiles of infections in the network of 5,000 nodes are 2.62% and 13.58%, whereas for the network of 80,000 nodes the 25th and 75th percentiles of infections are 6.56% and 12.94%. In particular, the smaller networks are more likely to see the epidemic end early by chance (final infection percentage close to 0). Similarly, population size also does not seem to greatly affect the number of at-risk sexual contacts individuals have on average. In Figure S12, we can see that the variation in number of at-risk contacts is much greater between relationship types and sexual activity strata than between network population sizes.

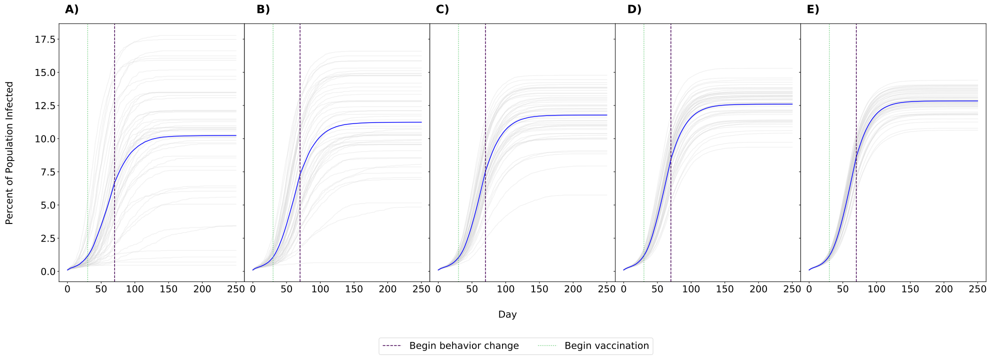

Fig. S11 Cumulative incidence with different population sizes. Panels indicate the percent of network infected with mpox after 250 days in a population of 5,000 nodes (A), 10,000 nodes (B), 20,000 nodes (C), 40,000 nodes (D), or 80,000 nodes (E). Grey lines denote individual simulations. The point-wise average is shown in blue. Vertical lines indicate the day of outbreak mitigation strategy initiation. The figure shows the runtime of 50 independent simulations of the scenario with behavior change and vaccination.

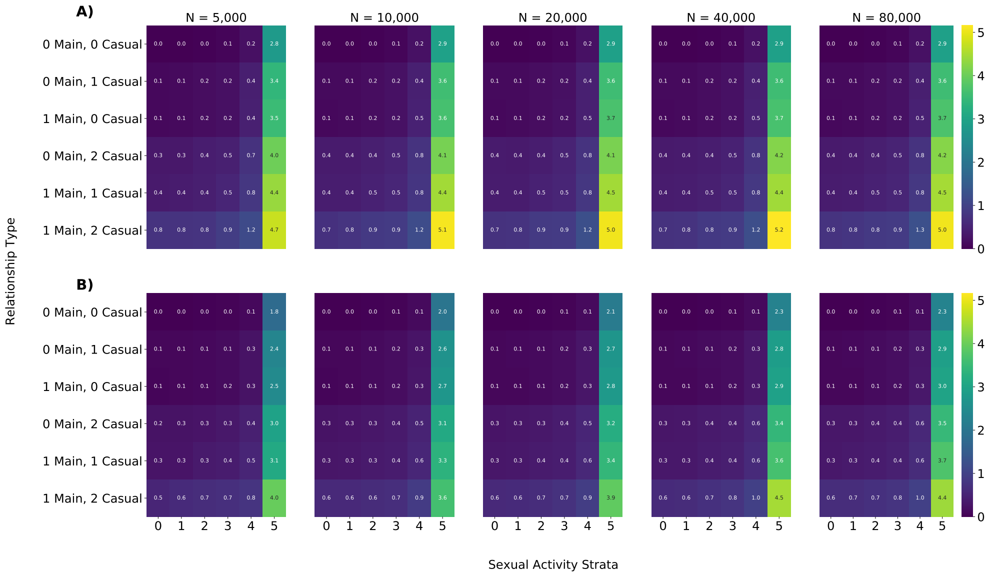

Fig. S12 Comparison of serodiscordant sexual interactions by relationship type and sexual activity strata Cells indicate the average number of serodiscordant sexual interactions individuals of a given relationship type and sexual activity stratum have over 250 days with no vaccination or behavior adaptation (Panel A) or vaccination and behavior adaptation only in the 25% of GBMSM most likely to have a one-time partner (Panel B). Rows indicate relationship type (preferred number of main and casual partners), while columns indicate sexual activity strata, or a node's daily probability of having a one-time partner.

We also calculated the effect of network size on the runtime of the algorithm. Figure S13 shows the runtime in seconds of 50 simulations at different network population sizes. As network size doubles, the runtime increases approximately 4-fold, yielding a $O(n^2)$ computational complexity. Additionally, algorithmic complexity does not differ greatly between running simulations with or without behavior change and vaccination.

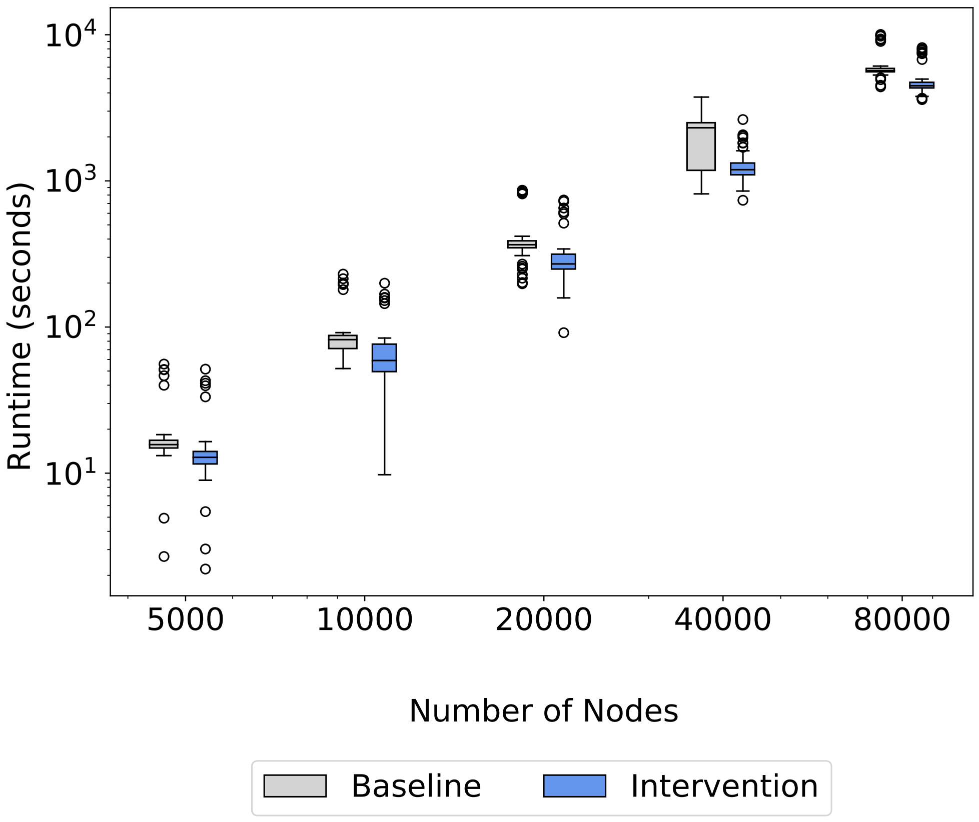

Fig. S13 Comparison of runtime for network populations The figure shows the runtime of 50 independent simulations of the baseline scenario without vaccination or behavior change, as well as the main scenario with behavior change and vaccination. All simulations are run for 250 time steps.

1.2.5 Network Dynamics and Structure

Due to the dynamic nature of the network, edge rewiring, or partner re-pairing, must be performed every time step. While the algorithm attempts to maintain each node's desired degree for main and casual partnerships, if there are no available partners when a relationship dissolves, those nodes must wait until another relationship dissolves to form new partnerships. Table S1 shows the average percentage of rewirings over 50 independent simulations, which do not happen in the same time step as the previous

partnership dissolved. Even for the smallest network we examined, fewer than 3% of the instances of rewiring could not occur immediately, demonstrating that this process nearly always occurs in the same time step, regardless of the overall size of the network. Therefore, we can be confident that the overall degree distribution for main and casual partnerships remains stationary.

Table S1 Percentage of delayed rewirings for main and casual partnerships

**Network Population Size**	**Main Partnerships**, Mean (SD)	**Casual Partnerships**, Mean (SD)
N = 5,000	0.03 (0.008)	2.54×10^{-5} (0.014×10^{-4})
N = 10,000	0.002 (0.002)	1.42×10^{-5} (4.82×10^{-5})
N = 20,000	1.15×10^{-5} (8.07×10^{-5})	1.93×10^{-6} (1.34×10^{-5})
N = 40,000	0 (0)	0 (0)
N = 80,000	0 (0)	1.28×10^{-6} (6.69×10^{-6})

To better understand the structure of the network, we examined different measures of network structure: transitivity, defined as the percentage of completed triangles (Figure S14); average clustering coefficient (Figure S15); the proportion of nodes contained in the largest connected component (LCC) (Figure S16); average node degree (Figure S17); and maximum degree in the network (Figure S18). Given that the network is dynamic, we look at cumulative edges over a 7-day period in the network. We compare these network summaries for different population sizes, between baseline models and models including outbreak mitigation strategies, and at different times throughout the outbreak. The outbreak mitigation strategies compared are that of the main result, with vaccination beginning on day 30 and behavior change beginning on day 70. The timings selected were day 28, before any vaccination or behavior change, and day 84, two weeks after the behavior change begins, in order to visualize the impact of the outbreak mitigation strategies on network structure.

In general, we find that on day 28, prior to vaccination/behavior change, the baseline and outbreak mitigation models are identical, which is as expected. After the initiation of vaccination/behavior change, summaries of network connectedness

decrease. This is true in both the baseline and outbreak mitigation models. In baseline models, this is due to the increased number of infected individuals as the infection spreads and, thus, the increased number of individuals beginning to comply with isolation recommendations. In the outbreak mitigation scenarios, the decrease in network connectedness is further pronounced by implementing behavioral changes.

Average transitivity, or the proportion of completed triangles in the graph, as well as the average clustering coefficient, the fraction of completed possible triangles involving a particular node and then averaged over all nodes, can be thought of as measures of local connectivity in the graph. In this context, they look at how frequently two partners have a partnership with the same third partner. We expect this to decline with increased network size when partnerships are defined by random chance, as in the case of this model, because there are more nodes with which to partner.

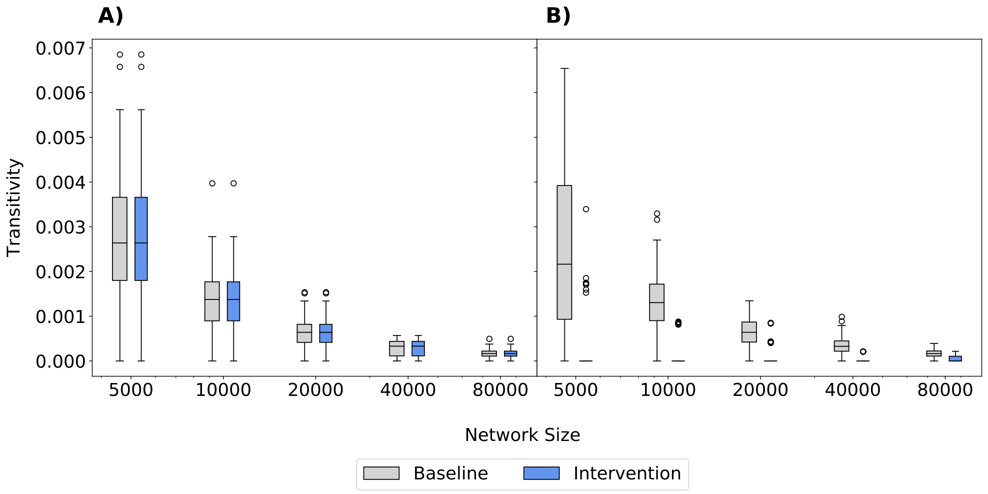

Fig. S14 Comparison of transitivity Panels show the transitivity of the network at day 28 (Panel A) and day 84 (Panel B) of the simulation. Results presented are for 50 independent simulations of the baseline (no vaccination/behavior change) and models with vaccination/behavior change.

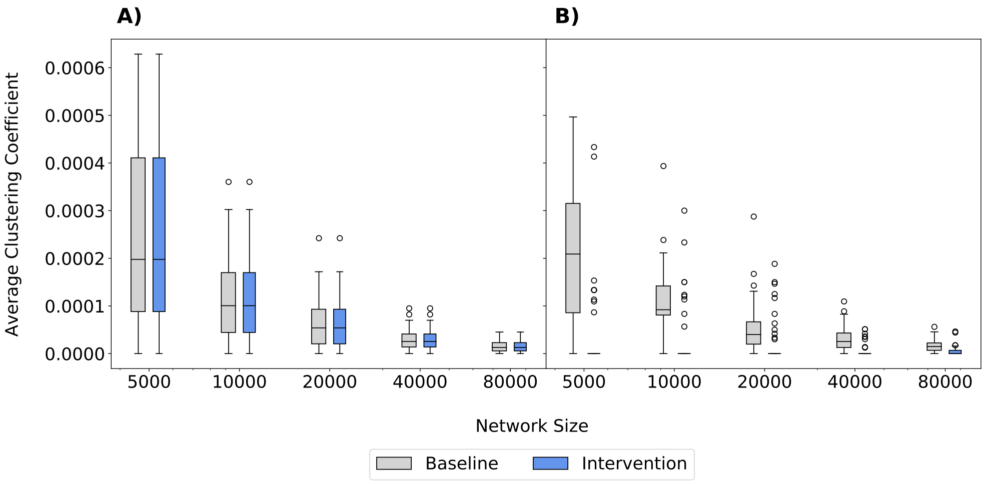

Fig. S15 Comparison of average clustering coefficient Panels show the average clustering coefficient of the network at day 28 (Panel A) and day 84 (Panel B) of the simulation. Results presented are for 50 independent simulations of the baseline (no vaccination/behavior change) and models including vaccination and behavior change.

The proportion of nodes contained in the LCC corresponds to a time-averaged dyanmic graph. In static graphs, the proportion of nodes in the LCC can be a rough indicator of the maximum proportion of nodes who could be infected by a disease-spreading process from a single source. Given that our network model is dynamic, this can be considered only as a snapshot in time and could be highly variable if, for example, a new edge forms between two connected components. However, the change in the proportion of nodes in the LCC after vaccination and behavior adaptation is still informative; it indicates an overall decline in the global connectedness of the graph, which indicates that it is less likely for an infection to become widespread over the network.

Average degree and maximum degree are summaries of node-level connectedness in the graph. In Figure S17, we see that, on average, the typical individual in our model has fewer than one partner over a single week. As expected, this highlights

how sparse the network is. Maximum degree looks at the most connected node in the graph; in a disease-spreading process, this person can be considered a 'super spreader' and is often someone whose behavior greatly impacts the outbreak. While we expect the maximum degree in a graph to increase slightly with the overall size of the graph, this is somewhat limited. The number of one-time partners an individual seeks on a particular day is defined by a geometric distribution, parameterized by their daily probability of having a one-time partner, which is unaffected by network size. Larger networks merely have more opportunities to see a very high degree node by random chance. It is important to highlight the change in maximum degree after vaccination and behavior adaptation; in this context, it demonstrates that the maximum number of nodes that could be infected by a single node over a week has declined.

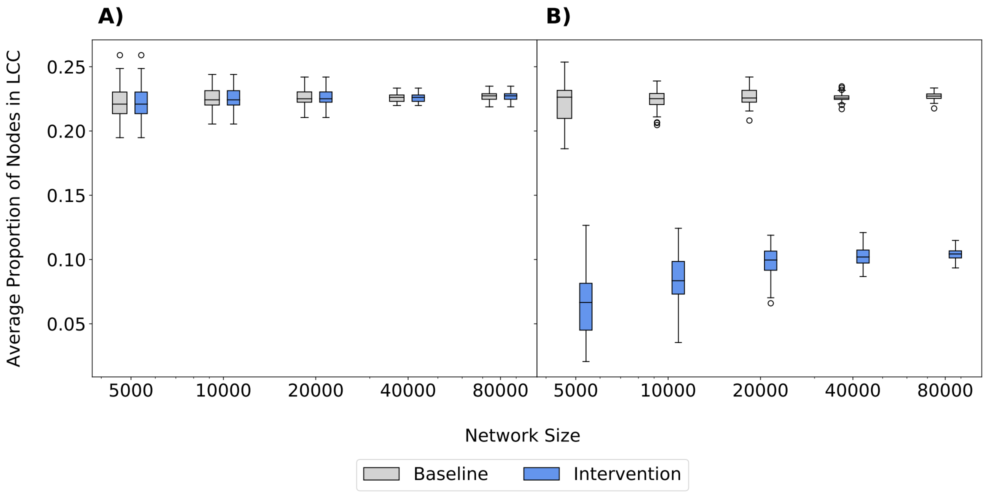

Fig. S16 Comparison of the proportion of nodes part of the largest connected component Panels show the proportion of nodes which are part of the largest connected component of the network at day 28 (Panel A) and day 84 (Panel B) of the simulation. Results presented are for 50 independent simulations of the baseline model (no vaccination/behavior change) and models including vaccination and behavior change.

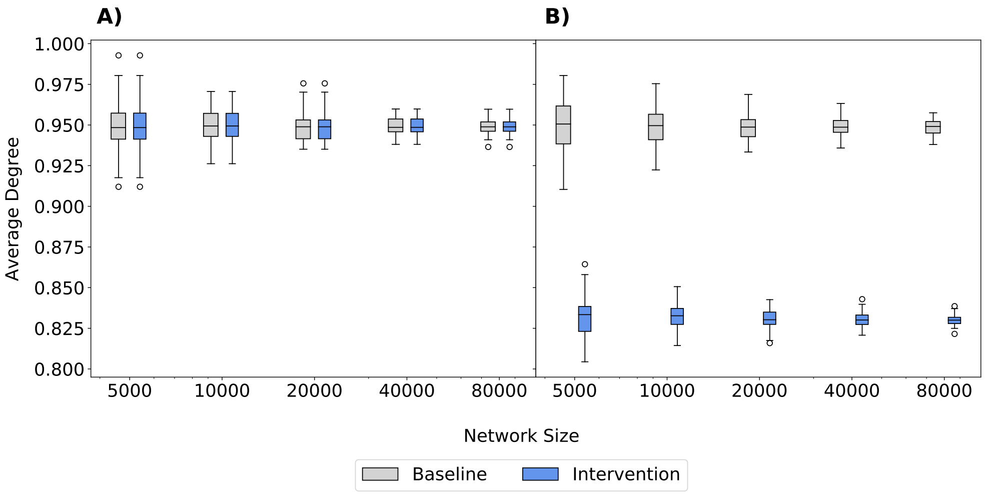

Fig. S17 Comparison of average degree Panels show the average degree of the network at day 28 (Panel A) and day 84 (Panel B) of the simulation. Results presented are for 50 independent simulations of the baseline model (no vaccination/behavior change) and models including vaccination and behavior change.

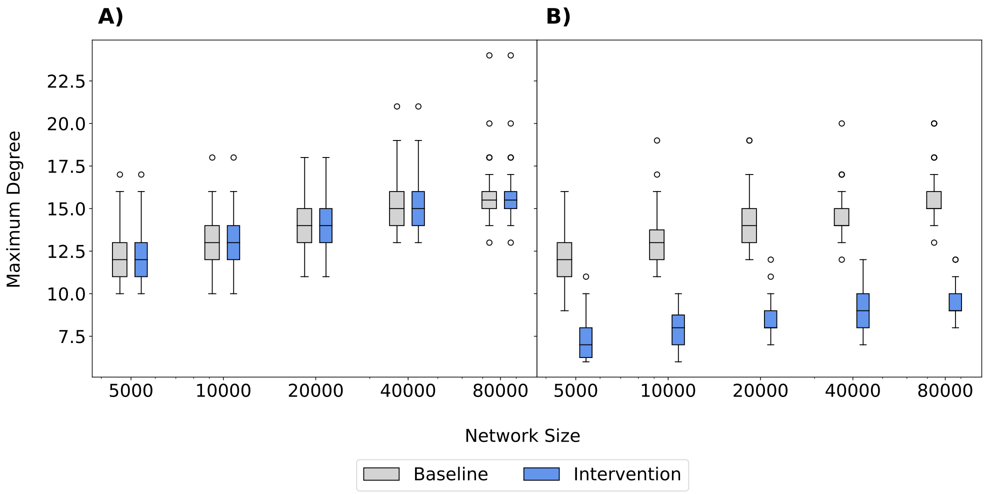

Fig. S18 Comparison of maximum degree Panels show the maximum degree of the network at day 28 (Panel A) and day 84 (Panel B) of the simulation. Results presented are for 50 independent simulations of the baseline model (no vaccination/behavior change) and models including vaccination and behavior change.

1.2.6 Simulation Stability

To ensure that enough simulations were used to have a stable estimate of the average number of final cumulative infections, we included plots of the mean cumulative infections as the number of simulations increases for different simulation scenarios in Figure S19.

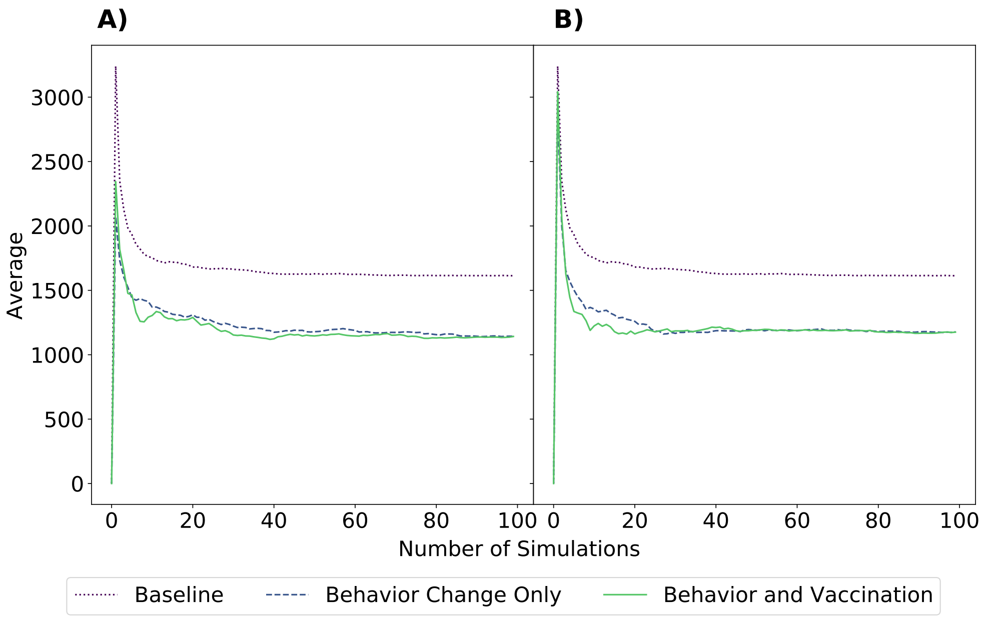

Fig. S19 Stability plots of average final infection counts Panels show the results for universal vaccination/behavior change (Panel A) and restricted vaccination and behavior change (Panel B). Results presented are for 100 independent simulations of the baseline model (no vaccination/behavior change) and models including vaccination and behavior change.

1.3 Simulation Algorithms

Algorithm 1 CreateGraph (Graph Initialization)

Require: Number of nodes N, Daily probability of one-time partnership formation $\pi_{o,k}$, Relationship type distribution rt_k

repeat

Assign relationship type for each node:

$$rt_k[x] \sim \text{Categorical}([0.471, 0.167, 0.074, 0.22, 0.047, 0.021])$$

Compute degree sequence of main and casual partners:

$$n_m[x] = \begin{cases} 0, & \text{if } rt_k[x] \in \{0, 1, 2\} \\ rt_k[x] - 2, & \text{otherwise} \end{cases}$$

$$n_c[x] = \begin{cases} 0, & \text{if } rt_k[x] \in \{0, 3\} \\ 1, & \text{if } rt_k[x] \in \{1, 4\} \\ 2, & \text{otherwise} \end{cases}$$

Assign sexual activity stratum:

$$strat_{o,k} \sim \text{Categorical}([0.19, 0.19, 0.19, 0.19, 0.19, 0.05])$$

Compute one-time degree sequence given sexual activity stratum:

$$n_o[x] \sim \text{Geometric}(1 - \pi_{o,k}[strat_{o,k}])$$

until $\sum n_m$, $\sum n_c$, and $\sum n_o$ are all even

Initialize graph G with N nodes

return $G, n_m, n_c, n_o, rt_k, strat_{o,k}$

Algorithm 2 init_relationships (Initialize Relationships in the Network)

Require: Graph G, degree sequences for one-time (n_o) main (n_m) and casual partnerships (n_c), average duration of main partnerships $rd_{m,e}$, and average duration of casual partnerships $rd_{c,e}$

Create stub lists for main D_m, casual D_c, and one-time partnerships D_o
for x in G **do**
    append $n_m[x] \cdot x$ to D_m
    append $n_c[x] \cdot x$ to D_c
    append $n_o[x] \cdot x$ to D_o
end for
Randomly shuffle D_o
for each pair in D_o **do**
    Create one-time relationship (u, v)
end for
Randomly shuffle D_m
for each pair in stub list of D_m **do**
    Create main relationship (u, v) with duration $\sim$ Geometric($rd_{m,e}$)
end for
Randomly shuffle D_c
for each pair in stub list of D_c **do**
    Create casual relationship (u, v) with duration $\sim$ Geometric($rd_{c,e}$)
end for
return G, main relationships, casual relationships, one-time relationships

Algorithm 3 update_onetime (Update One-Time Relationships)

Require: Graph G, One-time encounter probability $\pi_{o,k}$, Activity stratum $\pi_{o,k}$

```
Remove all existing one-time relationships
repeat
    for each node x ∈ G do
        Sample number of one-time partners:
```

$$n_o[x] \sim \text{Geometric}(1 - \pi_{o,k}[x])$$

```
    end for
until Total stubs ∑ n_o is even
Shuffle one-time stubs and randomly pair nodes to form relationships
for each pair (u, v) do
    Add one-time edge (u, v)
end for
return G, n_o
```

Algorithm 4 update_relationships (Update Main and Casual Relationships)

Require: Graph G

Remove expired relationships:
for each edge (u, v) in G **do**
  if Relationship duration has ended **then**
    Remove edge (u, v)
    if main relationship **then**
      Add u, v to list of nodes wanting a main partner, W_m, update $X_m[u] = v$, $X_m[v] = u$
    else if casual relationship **then**
      Add u, v to list of nodes wanting a casual partner, W_c, update $X_c[u] = v$, $X_c[v] = u$
    end if
  end if
end for
Form new main partnerships:
while $|W_m| > 1$ **do**
  Select node $x \in W_m$, find partner $y \notin X_m[x]$
  if y exists **then**
    Create main relationship (x, y) with duration $\sim$ Geometric($rd_{m,e}$)
    Remove x, y from W_m
  end if
end while
Form new casual partnerships:
while $|W_c| > 1$ **do**
  Select node $x \in W_c$, find partner $y \notin X_c[x]$
  if y exists **then**
    Create casual relationship (x, y) with duration $\sim$ Geometric($rd_{c,e}$)
    Remove x, y from W_c
  end if
end while
return Updated G

Algorithm 5 spread (Infection Spread in the Network)

Require: Graph G, Probability of infection β, Susceptible nodes S, Exposed nodes E, Infected nodes I, Contact probabilities π_m, π_c, π_o, Vaccine efficacy VE

for each infected node i in I **do**
    Determine compliance level for isolation
    Identify susceptible neighbors $N_i = \{j \mid j \in S\}$
    for each $j \in N_i$ **do**
        Determine contact probability π_m, π_c, π_o based on relationship type
        if Contact occurs and transmission succeeds ($U \sim \text{Uniform}(0,1) < \beta \cdot (1 - VE_j)$ **then**
            Add j to E and remove j from S
            Record infection source and time
        end if
    end for
end for
return S, E, I, infection sources and time

Algorithm 6 update_status (Update Infection Status)

Require: Susceptible nodes S, Exposed nodes E, Infected nodes I, Recovered nodes R, Infection times $t_{i,k}$, Exposure times $t_{e,k}$, Treatment delays T_d, Current step t

Update remaining time in exposed and infected states:
for each $x \in E$ **do** $t_{e,k}[x] \leftarrow t_{e,k}[x] - 1$
end for
for each $x \in I$ **do** $t_{i,k}[x] \leftarrow t_{i,k}[x] - 1$
end for
Identify individuals transitioning states
    $E \rightarrow I$: $E_{\text{new}} \leftarrow \{x \mid t_{e,k}[x] \leq 0\}$
    $I \rightarrow R$: $I_{\text{new}} \leftarrow \{x \mid t_{i,k}[x] \leq 0\}$
return S, E, I, R

Algorithm 7 vaccinate (Vaccination Process)

Require: Graph G, Vaccine availability at time t for each dose $V_{1,t}, V_{2,t}$, Vaccination probabilities for each dose π_{v_1}, π_{v_2}, Vaccine efficacy for each dose VE_1, VE_2

Identify eligible nodes for first dose: $W_1 = \{x \notin I \bigcup R, V[x] = 0\}$
Allocate vaccines: $V_1 \leftarrow \text{Sample}(W_1, \min(W_1, V_{1,t}))$
for each $x \in V_1$ **do**
    Set first dose received ($V[x] = 1$)
end for
Identify eligible nodes for second dose: $W_2 = \{x \mid V[x] = 1, \text{Time elapsed} \geq 28\}$
Allocate second doses: $V_2 \leftarrow \text{Sample}(W_2, \min(W_2, V_{2,t}))$
for each $x \in V_2$ **do**
    Set second dose received ($V[x] = 2$)
end for
Update vaccine efficacy: $VE[x] = VE_2$ if $V[x] = 2$, else VE_1
return Updated vaccine status

Algorithm 8 simulate (Run Simulation)

Require: Number of nodes N, Initial infections n_{init}, Infection probability β, Steps T, Vaccination/behavior change start T_{int}, Behavior change π_b, Isolation level iso, Vaccination delay T_v, Daily vaccine availability over time V_1, V_2

Initialize network G with N nodes using CreateGraph
Initialize infected nodes I
Generate exposure times $t_{e,k} \sim \mathcal{N}(7, 1)$ and infection times $t_{i,k} \sim \mathcal{N}(27, 3)$
Compute initial relationship structure using INIT_RELATIONSHIPS
for each time step $t = 1$ to T **do**
    if No exposed or infected nodes remain **then**
        break
    end if
    if $t = T_{int}$ **then**
        Apply behavior change π_b
    end if
    if $t \geq T_v$ **then**
        Administer vaccinations using VACCINATE
    end if
    Update infection status using UPDATE_STATUS
    Spread infection using SPREAD
    Update relationships using UPDATE_ONETIME and UPDATE_RELATIONSHIPS
    Record epidemic dynamics: S, E, I, R
end for
return S, E, I, R

2 References

References

[1] CDC: Mpox Vaccine Administration in the U.S. Available from: https://www.cdc.gov/poxvirus/mpox/response/2022/vaccines_data.html.

- Note: This information is no longer available on the CDC website. The data used for this analysis is available on GitHub at https://github.com/onnela-lab/mpox-model as the file *mpox_vax_coverage_data.xlsx* or via the Wayback Machine (for July 10, 2023 rather than the date the data was pulled originally on July 11, 2023) at https://web.archive.org/web/20230710132210/https://www.cdc.gov/poxvirus/mpox/response/2022/vaccines_data.html

[2] CDC: JYNNEOS Vaccine Coverage by Jurisdiction. Available from: https://www.cdc.gov/poxvirus/mpox/cases-data/mpx-jynneos-vaccine-coverage.html.

[3] Owens LE, Currie DW, Kramarow EA, Siddique S, Swanson M, Carter RJ, et al. JYNNEOS Vaccination Coverage Among Persons at Risk for Mpox — United States, May 22, 2022–January 31, 2023. MMWR Morbidity and Mortality Weekly Report. 2023;72(13):342–347. https://doi.org/10.15585/mmwr.mm7213a4.

[4] Deputy NP, Deckert J, Chard AN, Sandberg N, Moulia DL, Barkley E, et al. Vaccine Effectiveness of JYNNEOS against Mpox Disease in the United States. New England Journal of Medicine. 2023;388(26):2434–2443. https://doi.org/10.1056/nejmoa2215201.

[5] Pischel L, Martini BA, Yu N, Cacesse D, Tracy M, Kharbanda K, et al. Vaccine effectiveness of 3rd generation mpox vaccines against mpox and disease severity: A systematic review and meta-analysis. Vaccine. 2024;42(25):126053. https://doi.org/10.1016/j.vaccine.2024.06.021.

[6] Ponce L, Linton NM, Toh WH, Cheng HY, Thompson RN, Akhmetzhanov AR, et al. Incubation Period and Serial Interval of Mpox in 2022 Global Outbreak Compared with Historical Estimates. Emerging Infectious Diseases. 2024;30(6):1173–1181. https://doi.org/10.3201/eid3006.231095.
